# Environmental assessment of antibiotic toxicity under climate change–related temperature and pH scenarios using *Danio rerio*

**DOI:** 10.1007/s10695-026-01750-9

**Published:** 2026-07-31

**Authors:** Bárbara S. Diogo, Sara Rodrigues, Oksana Golovko, Sara C. Antunes

**Affiliations:** 1 Instituto de Ciências Biomédicas de Abel Salazar, Universidade Do Porto, Rua de Jorge Viterbo Ferreira, 228, 4050-313 Porto, Portugal; 2 CIIMAR/CIMAR LA, Centro Interdisciplinar de Investigação Marinha E Ambiental, Universidade Do Porto, Terminal de Cruzeiros do Porto de Leixões, 4450-208 Matosinhos, Portugal; 3 Departamento de Biologia, Faculdade de Ciências, Universidade Do Porto, Rua Do Campo Alegre S/N, 4169-007 Porto, Portugal; 4 Centro de Biologia Molecular E Ambiental (CBMA)/Rede de Investigação Aquática (ARNET), Departamento de Biologia, Escola de Ciências da Universidade Do Minho, Braga, Portugal; 5 Instituto de Ciência e Inovação Para a Bio-sustentabilidade (IB-S), Escola de Ciências da Universidade Do Minho, Braga, Portugal; 6 Departamento de Ciências Aquáticas e Avaliação, Universidade Sueca de Ciências Agrícolas (SLU), 75007 Uppsala, Sweden

**Keywords:** Sulfamethoxazole, Trimethoprim, Mixture, Zebrafish, Environmental stressors, Ecotoxicity

## Abstract

Climate change induced temperature and pH shifts can alter aquatic environments, potentially intensifying antibiotic toxicity, disrupting the physiological balance of aquatic organisms. Few studies have examined combined effects of these abiotic environmental stressors and antibiotics on aquatic ecosystems in a potential climate change scenario. This study evaluated the toxicity of environmentally relevant concentrations of sulfamethoxazole (150 µg SMX/L), trimethoprim (30 µg TRIM/L), and their mixture (MIX: 150 µg SMX/L + 30 μg TRIM/L) in *Danio rerio* under two scenarios: standard (26 °C, pH 7.5) and climate changed (28 °C, pH 9.0). A multi-biomarker approach was performed to evaluate oxidative stress, lipid peroxidation, neurotransmission, energy metabolism, and DNA damage. Integrative assessment of an organism’s health showed that increasing pH and temperature intensified SMX and TRIM toxicity, shifting from marginally to highly toxic. Meanwhile, MIX toxicity increased from marginally to moderately toxic. Under standard conditions, SMX and TRIM caused major alterations (affecting antioxidant/detoxification mechanisms), while MIX induced severe alterations in *D. rerio*'s health status. Under a climate-changed scenario, all antibiotics led to severe alterations (lipid peroxidation, DNA damage, and energy metabolism disruption). These findings suggest that under a climate-changed scenario (higher temperature and pH increase), both individually and in mixtures, antibiotic toxicity leads to more severe effects on *D. rerio*. Given this, it is crucial to consider environmental factors when assessing antibiotic toxicity, as shifts in temperature and pH can significantly amplify their harmful effects. Understanding these interactions is essential for developing effective regulatory and conservation strategies to safeguard aquatic ecosystems from long-term ecological imbalances.

**Supplementary Information:**

The online version contains supplementary material available at https://doi.org/10.1007/s10695-026-01750-9.

## Introduction

Climate change significantly impacts aquatic ecosystems, particularly through alterations in environmental conditions such as water body temperature and pH (Bethke et al. 2023; IPCC 2023). These abiotic factors are essential for maintaining ecological balance and the health status of aquatic ecosystems (Kazmi et al. 2022; Marium et al. 2023; Suter et al. 2023). Long-term shifts in temperature and pH, driven by ongoing climate change, are altering the environmental baseline (e.g., tolerance ranges and adaptive capacities of aquatic species) for many aquatic systems. Rather than temporary fluctuations, these changes can establish new and persistent conditions that disrupt the optimal physiological range for numerous species, compromising their survival and overall ecosystem functioning (Prakash 2021; Thomas et al. 2022). According to projections by the Intergovernmental Panel on Climate Change (IPCC), a continuous rise in global temperatures and changes in pH are expected, factors that may further compromise the stability of aquatic habitats, making them more vulnerable to a variety of environmental stressors (IPCC 2023; Rose et al. 2023). These environmental stressors, although often studied in isolation, co-occur and interact in complex ways that may amplify their individual and combined effects (Bethke et al. 2023; Marium et al. 2023). In freshwater ecosystems, which remain understudied compared to marine environments (Rose et al. 2023), projections indicate substantial changes in key abiotic factors, such as temperature and pH. According to the IPCC (2023), and Stockwell et al. (2021), global surface temperatures are expected to rise by 1.0 to 1.8 °C, potentially up to 2 °C, by the end of the century under low-emission scenarios. In parallel, several studies suggest a trend toward freshwater alkalinization, with pH levels rising due to anthropogenic influences and biogeochemical shifts (INAG, 2011a, 2011b, 2011c; Kaushal et al. 2013, 2018; Pinto et al. 2025). These altered conditions have already been associated with physiological and ecological impacts in freshwater organisms, including *D. rerio* (e.g., Bethke et al. 2023). Despite these projections, most experimental studies still focus on one or two stressors, often addressing either temperature or pH individually, and rarely in combination with chemical contaminants. Among these, antibiotics are frequently detected in freshwater ecosystems and have received increasing attention due to their continuous input, pseudo-persistence, and biological activity even at low concentrations. Two of the most widely used antibiotics are sulfamethoxazole (SMX) and trimethoprim (TRIM), which are included in the two last EU Water Framework Directive Watch List to monitor substances of potential ecological concern (Cortes et al. 2020), highlighting their environmental relevance. Moreover, the rising detection of these compounds, and their mixture (MIX), is largely attributable to their extensive application in human and veterinary medicine, aquaculture, and agriculture (Carvalho and Santos 2016). Although typically detected in surface waters at ng/L concentrations, these compounds have been reported at µg/L levels in specific scenarios, including wastewater effluents, untreated sewage-impacted areas, and regions of intensive pharmaceutical use, with concentrations reaching up to 150 µg/L for SMX and 30 µg/L for TRIM (Kairigo et al. 2020; Khan et al. 2013). This widespread presence highlights a significant knowledge gap regarding the effects (e.g., synergistic, antagonist, or additive effect) of multiple stressors under realistic future scenarios.

To address these gaps, it is essential to advance our understanding of how combined stressors, particularly those associated with climate change and chemical contamination, affect aquatic ecosystems. While each stressor independently poses substantial risks, their concurrent occurrence can lead to unpredictable and potentially amplified interactions, impacting both organismal health and ecosystem stability (Danylchuk et al. 2023). These combined effects remain poorly understood, especially in freshwater ecosystems, which are experiencing substantial environmental changes and facing serious threats to their biodiversity and ecological function (Jesus et al. 2018). Freshwater ecosystems provide essential services, including water purification, carbon sequestration, and habitats for diverse species (Vári et al. 2022). However, they are increasingly threatened by rising global temperatures, pH fluctuations, and chemicals, such as antibiotics (Gomes 2024; IPCC 2023). The use of diverse and sensitive biological endpoints enhances the detection of how multiple stressors influence aquatic organisms at different levels of biological organization (Abdallah et al. 2024). The integration of multiple biological endpoints and biomarkers (e.g., oxidative stress, lipid peroxidation, cholinergic neurotransmission, energy metabolism, and DNA damage) provides a detailed understanding of physiological and behavioral responses, contributing to a more comprehensive evaluation of both short- and long-term ecological impacts (El-SiKaily and Shabaka 2024).

Given the global importance of aquatic ecosystems, understanding abiotic and pollutant interactions is vital for developing strategies to mitigate the compounded effects of climate change and pollution. Effective management policies must integrate scientific findings to address these interconnected threats, ensuring the sustainability of aquatic habitats and the health of the communities reliant on them (Rinke et al. 2025). Thus, the objective of this study is to investigate the combined impact of antibiotics and climate change, specifically increased temperature and pH, on freshwater ecosystems, using *Danio rerio* as a model species. Environmentally relevant concentrations of SMX (150 µg/L), TRIM (30 µg/L) (Kairigo et al. 2020; Khan et al. 2013), and their mixture (MIX = 150 µg/L of SMX + 30 µg/L of TRIM) were tested under two different scenarios: a standard (normal temperature and pH) and a climate-changed scenario (elevated temperature and pH). A multi-biomarker analysis and an integrative assessment of the organism’s health were applied to evaluate the effects on the *D. rerio*’s biological health, offering valuable insights into the combined impact of climate change factors and antibiotic exposure as multiple stressors.

## Material and methods

### Chemicals, stock solutions, and treatments

Sulfamethoxazole (SMX; CAS: 723–46-6; molecular weight 253.28 g/mol; purity ≥ 98%) and trimethoprim (TRIM; CAS: 738–70-5; molecular weight 290.3 g/mol; purity ≥ 98.5%) were purchased from Sigma-Aldrich. Stock solutions of SMX (100 mg/L) and TRIM (50 mg/L) were prepared by dissolving the antibiotics in dechlorinated tap water. The tested concentrations, 150 µg/L for SMX and 30 µg/L for TRIM, were selected to represent worst case but environmentally relevant scenarios in surface freshwater ecosystems impacted by untreated sewage and high pharmaceutical use (Kairigo et al. 2020; Khan et al. 2013). Additionally, a mixture of SMX and TRIM (MIX = 150 µg SMX/L + 30 µg TRIM/L) was tested to reflect potential co-occurrence under these conditions (Carvalho and Santos 2016; Kairigo et al. 2020; Khan et al. 2013).

Two scenarios were designed to investigate the antibiotic effects in a climate change context. A standard scenario simulated optimal conditions for *Danio rerio*, maintaining a temperature of 26 ± 1 °C and a neutral pH (7.5 ± 0.5), representative of the standard guideline recommendations, and the water laboratory system conditions (OECD 2000). A climate-changed scenario combined the effects of increased temperature and pH, based on the worst-case conditions previously identified in previous studies, integrating both stressors simultaneously to evaluate their combined effects under a realistic climate change scenario. The temperature was increased by 2 °C above the standard temperature (28 °C; based on global warming projections from the Intergovernmental Panel on Climate Change and Climate Action Tracker), which estimates an average global surface temperature rise of 1.0–1.8 °C (maximum ~ 2 °C) by 2081–2100 under low greenhouse gas emissions scenarios (IPCC 2023; Stockwell et al. 2021), and the toxic effects on *D. rerio* health reported by Diogo et al. (2025c). The pH was adjusted to 9.0, reflecting alkaline projections for freshwater ecosystems (INAG, 2011c, 2011a, 2011b; Kaushal et al. 2013, 2018; Pinto et al. 2025), and the toxic effects on *D. rerio* health reported by Diogo et al. (2025b).

### Antibiotic quantifications

For the quantification of SMX, TRIM, and MIX measured concentrations (Table 1), a volume of 50 mL of water was randomly collected from a replicate of each treatment from all exposure aquaria, at the beginning of the assay (0 h), and before the renewal of the medium (48 h) to confirm that antibiotic levels remained within an acceptable range of nominal concentrations, supporting their stability over the standard renewal period established by the guideline (OECD 2000). Water samples were immediately stored in the dark and frozen at − 20 °C until further quantification of each antibiotic. Before analysis, samples were filtered through a 0.22-µm regenerated cellulose syringe filter, and 1 mL aliquots was spiked with 10 ng of internal standards (trimethoprim-13c-d3 and sulfamethoxazole-d4). The samples were analyzed using a DIONEX UltiMate 3000 UPLC system (ultra-high pressure liquid chromatography system—Thermo Scientific, Waltham, MA, USA) coupled to triple quadrupole mass spectrometer (MS/MS) (TSQ QUANTIVA, Thermo SCIENTIFIC, Waltham, MA, USA). An Acquity UPLC BEH-C18 column (100 mm × 2.1 mm, 1.7 µm) was used. Injection volume was 10 µL, and compounds were ionized using heated electrospray ionization (H-ESI) in positive mode (spray voltage 3500 V). Nitrogen (> 99.999% purity) was used as sheath, auxiliary, and sweep gas (50, 15, and 2 arbitrary units, respectively), with a vaporizer temperature of 400 °C and capillary temperature of 325 °C. The mobile phase consisted of Milli-Q water with 5 mM ammonium acetate and acetonitrile, with a flow rate of 0.5 mL/min over a 15-min run. Instrument control and method optimization were performed using Xcalibur software, and data were processed in TraceFinderTM 3.3 software. Linearity was verified from 0.1 to 1000 ng/mL, and the limit of quantification (LOQ) was defined as one-quarter of the lowest calibration point with a relative standard deviation of the average response factor was < 30%. LOQs were 0.25 µg/L for SMX and 0.39 µg/L for TRIM. The method’s precision was verified through repeatability by preparing all samples in triplicate. No traces of the studied compounds were detected in the control samples.

**Table 1:** Results of the measured concentrations of control group (CTL), sulfamethoxazole (SMX), trimethoprim (TRIM), and mixture (MIX) in water samples collected at the beginning of the assay (0 h), and before the renewal of the medium (48 h). Physical and chemical parameters measured during chronic exposure were also presented (*n* = 3; mean ± SD). *Stands for established quality criteria of water quality parameters (pH, temperature and dissolved oxygen concentration) under standard scenario (OECD 2000)

**Experimental scenarios**		**Measured concentrations** (mg/L)		**pH**	**Temp.** (ºC)	**O**_**2**_ (mg/L)	**Nitrites** (mg/L)	**Ammonium** (mg/L)
**Scenarios**	**Treatments** (nominal concentrations mg/L)	0 h	48 h	6.5 to 8.5 ± 0.5*	21 to 25 ± 2 ºC*	> 60%*		
**Standard** 26 °C pH 7.5	CTL (SMX = 0.0 TRIM = 0.0)	SMX = 0.0 TRIM = 0.0	SMX = 0.0 TRIM = 0.0	7.56 ± 0.04	26.3 ± 0.15	90.2 ± 2.03	0.35 ± 0.22	0.50 ± 0.31
	SMX (150.0)	110.6 ± 13.9	88.0 ± 13.1	7.53 ± 0.01	26.3 ± 0.20	93.3 ± 1.16	0.26 ± 0.18	0.20 ± 0.04
	TRIM (30.0)	31.8 ± 4.6	29.3 ± 9.0	7.54 ± 0.04	26.5 ± 0.21	93.6 ± 2.12	0.22 ± 0.14	0.26 ± 0.21
	MIX (SMX = 150.0 TRIM = 30.0)	SMX = 132.0 ± 6.1 TRIM = 34.8 ± 2.1	SMX = 104.9 ± 3.6 TRIM = 33.6 ± 1.7	7.55 ± 0.04	26.8 ± 0.11	91.5 ± 2.66	0.32 ± 0.20	0.27 ± 0.14
**Changed** 28 °C pH 9.0	CTL (SMX = 0.0 TRIM = 0.0)	SMX = 0.0 TRIM = 0.0	SMX = 0.0 TRIM = 0.0	9.09 ± 0.07	28.4 ± 0.06	90.2 ± 1.92	0.28 ± 0.19	0.15 ± 0.10
	SMX (150.0)	80.0 ± 5.3	69.3 ± 6.0	9.02 ± 0.01	28.6 ± 0.15	92.6 ± 2.16	0.25 ± 0.10	0.39 ± 0.32
	TRIM (30.0)	33.7 ± 5.7	33.6 ± 1.8	9.05 ± 0.02	28.6 ± 0.21	90.6 ± 3.17	0.29 ± 0.20	0.35 ± 0.26
	MIX (SMX = 150.0 TRIM = 30.0)	SMX = 149.3 ± 10.9 TRIM = 37.9 ± 0.5	SMX = 144.9 ± 6.9 TRIM = 35.4 ± 3.8	9.06 ± 0.03	28.5 ± 0.17	92.4 ± 0.75	0.31 ± 0.21	0.42 ± 0.12

### Test organism, quarantine, and chronic exposure

Zebrafish juveniles (2 months old) were obtained from a laboratory broodstock and reared in an aquarium under controlled conditions (16 h light/8 h dark; 26 ± 1 °C) in laboratory-certified facilities at CIIMAR – Interdisciplinary Centre of Marine and Environmental Research (Matosinhos, Portugal). The acclimation period (3 weeks) was conducted in 60-L tanks with continuously aerated, dechlorinated tap water, under standard, controlled conditions of photoperiod (16 h light/8 h dark), temperature (26 ± 1 °C), and pH (7.5 ± 0.5). *D. rerio* juveniles were fed daily ad libitum with commercial zebrafish food (Zebrafeed 400–600 µm by Sparos) and were considered healthy and suitable for the assay, as no signs of disease or mortality were observed. Additionally, water quality parameters, including conductivity (267 ± 19 µS/cm) and dissolved oxygen (8.1 ± 0.2 mg/L), were monitored weekly using a multiparameter probe (Multi 3630 IDS SET F). Ammonium (0.31 ± 0.17 mg/L) and nitrite (0.28 ± 0.19 mg/L) concentrations were measured using a bench photometer (Spectroquant Multy Colimeter) from water samples taken from all aquariums (OECD 2000).

After the acclimation period, a chronic assay for 28 days was performed, according to the OECD Fish, Juvenile Growth Test, guideline No. 215 (OECD 2000) and two scenario conditions (standard *vs*. climate-changed), previously described. *D. rerio* juveniles (1.65 ± 0.01 cm; 0.070 ± 0.002 g) were assigned to twenty-four 2 L glass aquaria randomly distributed in the exposure room (3 aquaria per treatment, each one with 6 fish; a total of 144 fish). Every 48 h, fish were fed (Zebrafeed 400–600 µm by Sparos), and the exposure medium was 80% renewed according to the guideline recommendations (OECD 2000). A semi-static exposure design was adopted to maintain water quality and ensure relatively stable exposure concentrations throughout the assay, in line with common practices in chronic ecotoxicological tests. The experimental protocol was reviewed and approved by the CIIMAR Animal Welfare Body (CIIMAR-ORBEA 30–2019) and subsequently authorized by the Portuguese General Directorate for Food and Veterinary Medicine (DGAV; authorization no. 008592, issued on 20 May 2020).

### Fish sacrifice, biological sampling, and biomarker assessment

At the end of the exposure period (28 days), the organisms were euthanized by immersion in an ice-cold water bath (≤ 4 °C as rapid cooling as a pre-euthanasia step), followed by decapitation once opercular movements ceased and swimming ability was lost (Wilson et al. 2009). This method was deemed effective, swift, and minimally stressful for the fish, providing consistent and irreversible euthanasia while preserving tissue integrity (Wilson et al. 2009). All euthanasia and animal handling procedures were conducted under the approval of the CIIMAR Animal Welfare Body (CIIMAR-ORBEA 30–2019) and the Portuguese General Directorate for Food and Veterinary Medicine (DGAV; authorization no. 008592). AVMA guidelines (AVMA 2020) were considered as complementary international guidance regarding good euthanasia practices. All procedures involving euthanasia and animal handling were performed by FELASA-certified researchers (categories B and C), in accordance with EU and national regulatory requirements.

After euthanasia, the organisms were measured and weighed. Two fish whole bodies per aquarium/replicate were used to evaluate antioxidant defense and detoxification mechanisms (superoxide dismutase (SOD), catalase (CAT), glutathione peroxidase (GPx), glutathione reductase (GRed), glutathione S-transferases (GSTs) activities, and glutathione (GSH) levels), and for lipid peroxidation, measured as thiobarbituric acid reactive substances (TBARS) levels. Additionally, two fish whole bodies were used to evaluate energetic metabolism, one for lactate dehydrogenase (LDH) activity and the other for cellular energy allocation (CEA) analysis. The CEA assessment included measurements of available energy (Ea), determined by quantifying carbohydrates, lipids, and total protein contents, and energy consumed (Ec), evaluated through electron transport system (ETS) activity. For cholinergic neurotransmission analysis, one fish head per aquarium/replicate was used to determine acetylcholinesterase (AChE) activity. All biological samples (bodies and heads) were immediately frozen at − 80 °C and stored until biochemical determinations.

The enzymatic activities of SOD, CAT, GPx, GRed, GSTs, GSH content, and TBARS levels were determined in homogenates prepared with 2.5 mL of phosphate buffer (50 mM, pH 7.0) containing 0.1% Triton X-100, followed by centrifugation at 15,000 rpm (Hettich® MIKRO 200/200R) for 10 min at 4 °C. For CEA assessment, samples were homogenized in 1 mL of ice-cold phosphate buffer (50 mM, pH 7.0) and subsequently centrifugated at 10,000 rpm for 5 min at 4 °C. LDH activity was measured in 2.5 mL of TRIS/NaCl buffer (pH 7.2) and centrifuged at 6000 rpm for 3 min at 4 °C, while AChE activity was determined in 1.5 mL of phosphate buffer (0.1 M, pH 7.2) after centrifugation at 6000 rpm for 5 min at 4 °C. All the biomarkers were normalized by protein content, except the CEA biomarkers, which were expressed by mJ/mg of fresh weight. The protein content was quantified spectrophotometrically (wavelength 595 nm) and adapted to microplates using γ-globulin as a standard (Bradford 1976). Further details of the methodology used for these biomarker determinations are provided in Table S1.

Gills from one random fish per replicate were sampled and immediately processed to evaluate the genetic damage index (GDI). The comet assay was performed following the protocol described by Rodrigues et al. (2016), where gills were homogenized in 1 mL of refrigerated phosphate-buffered saline (PBS; pH 7.4) to obtain the cell suspension and centrifuged at 200 g for 5 min at 4 °C. A six-gel-per-slide system was employed, based on the model developed by Shaposhnikov et al. (2010) and detailed by Rodrigues et al. (2016). Each microgel (with 6 μL) was placed on glass microscope slides pre-coated with 1% normal melting point agarose (NMPA), arranged in two rows of three (three groups of two replicates) without coverslips. After the procedure, slides were stored in light-protected boxes until observation. DNA damage was quantified using a Nikon Eclipse Ci fluorescence microscope at 600 × magnification. For each sample (i.e., replicate), 100 nucleoids were analyzed and classified into five categories (0 to 4) based on the tail and head intensity, following Rodrigues et al. (2016). As positive controls, cells from control animals were exposed to 50 μM H_2_O_2_ for 5 min. The GDI was calculated using the method described by Azqueta and Collins (2011), and the results were expressed in arbitrary units on a scale of 0–400, based on 100 scored nucleoids.

### Statistical analyses

Data for all biomarkers were tested for normality using the Shapiro–Wilk test and for homogeneity using Levene’s test. Before statistical analysis, the data for SOD activity were transformed (log(x) + 1) to meet ANOVA assumptions. To evaluate the combined effects of each antibiotic (SMX, TRIM, and MIX) in the two scenarios (standard and climate-changed) on *D. rerio*, a two-way ANOVA was conducted. To assess differences between each antibiotic treatment and the respective control within each scenario, Dunnett’s test was performed. All statistical analyses were carried out with SPSS Statistics v29, using a significance level of *α* = 0.05.

#### Ecotoxicological assessment

To classify the ecotoxicity classes of each treatment regarding the impacts on *D. rerio* performance, the percentage of effects of each biomarker was calculated for each scenario tested (relative to the respective control group), according to the description by Rodrigues et al. (2022), with some adaptations (i.e., determination of percentage of effect). Ecotoxicity scores were assigned based on the 10%, 50%, and 90% effect levels (i.e., percentage of effects), following the methodology outlined by Rodrigues et al. (2022) (Table S3), and five ecotoxicity classes were defined (non-toxic, slightly toxic, marginally toxic, moderately toxic, highly toxic). These results were used to assess the toxic effects of each antibiotic under the two scenarios (Table S3).

#### Biological health status

Biomarker Response Index (BRI) was proposed as a method to assess the biological health status of organisms exposed to various stressors (Li et al. 2019). The BRI evaluates the extent of alterations in biomarker responses in stressed organisms relative to the baseline responses observed in a control group under non-stress conditions. BRI for each scenario tested was calculated according to Li et al. (2019) and Piva et al. (2011) and the *D. rerio* biological health status could be classified as negligible, moderate, major, or severe alterations based on the calculated BRI (Hagger et al. 2008; Table S4).

## Results and discussion

### Assessment of water quality

Throughout the exposure period (28 days), different water quality parameters, including conductivity, dissolved oxygen, ammonium, and nitrites, were maintained within the specified quality standards outlined by OECD (2000), as detailed in Table 1. Additionally, no mortality occurred during the assay, adhering to guideline criteria that require mortality in the control group to remain below 10%. No statistically significant alterations were observed in the specific growth rate of the organisms after antibiotic exposure across the tested temperature and pH scenarios (Table S2).

### Standard temperature and pH scenario

**Fig. 1:**
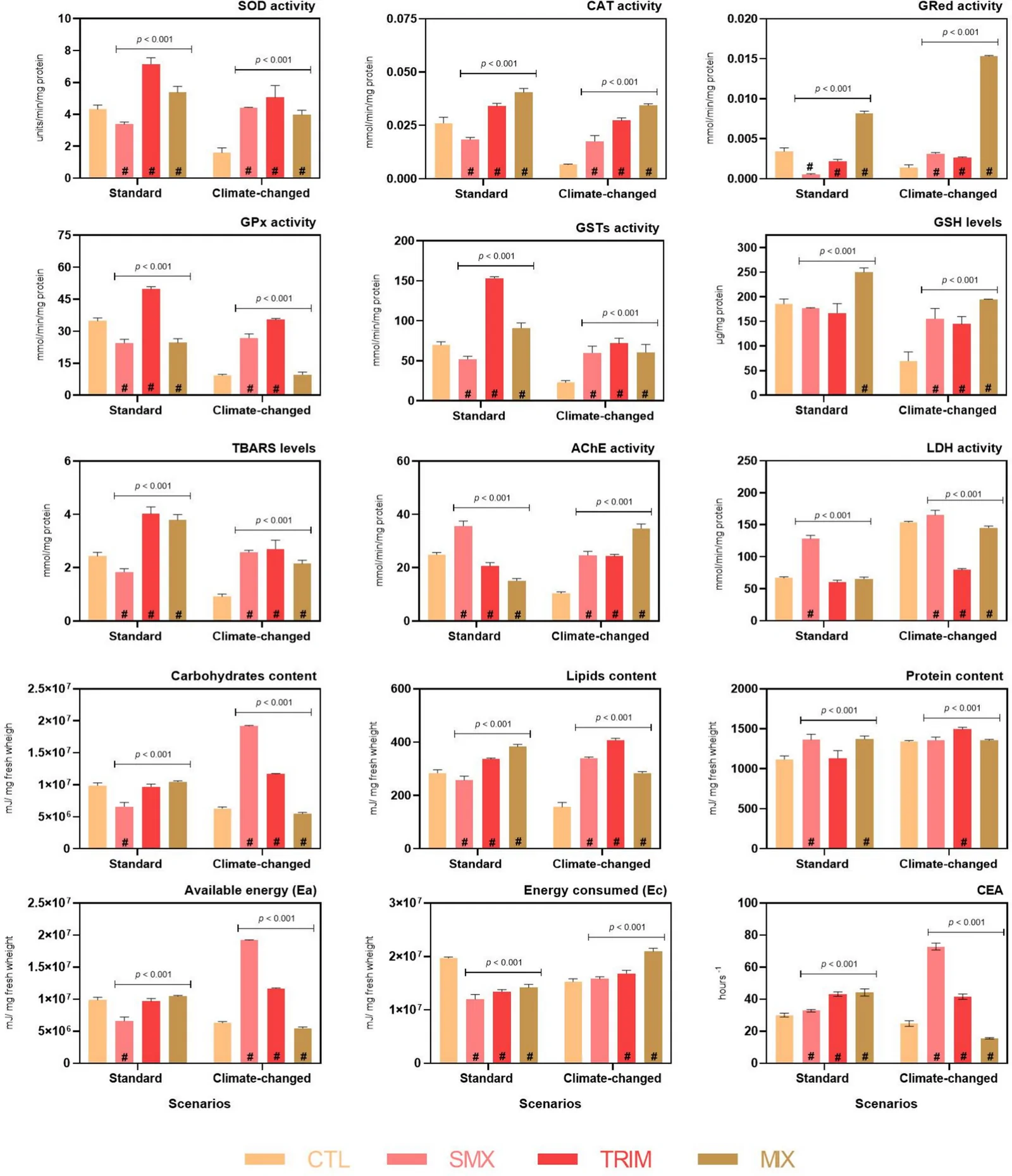
Results of biochemical markers of *Danio rerio* after chronic exposure (28 days) to the control group (CTL, without antibiotics), sulfamethoxazole (150 µg SMX/L), trimethoprim (30 µg TRIM/L), and mixture (MIX = 150 µg SMX/L + 30 µg TRIM/L) under standard (26 °C and pH 7.5) and climate-changed (28 °C and pH 9.0) scenarios. Data are expressed as mean ± standard error bars. Significant *p* levels correspond to the interaction effects between antibiotics and environmental scenarios tested (two-way ANOVA; Table S2). Number sign (#) discriminates significant differences between the control group and antibiotic treatments (Dunnett’s test; *p* < 0.05)

The results of *D. rerio* biochemical markers after exposure to environmentally relevant concentrations of SMX, TRIM, and their MIX under standard temperature and pH scenario (26 °C and pH 7.5) are shown in Fig. 1. Two-way ANOVA results (main effects and interactions) are presented in Table S2. Significant interactions were observed between antibiotic concentrations and standard environmental factors (temperature and pH) for all evaluated biochemical markers (Fig. 1; Table S2). Under the standard temperature and pH, SMX significantly reduced the activity of all antioxidant defense and detoxification mechanisms (SOD, CAT, GRed, GPx, and GSTs activities), as well as lipid peroxidation (measured through TBARS levels). Additionally, no significant changes in GSH levels were observed in *D. rerio* following exposure to SMX. These results suggest that SMX may induce a disruption in oxidative homeostasis, particularly by impairing the antioxidant defense system, as evidenced by the broad suppression of redox-related responses (Fig. 1). In contrast, under the same pH and temperature scenario, TRIM caused a significant increase in the activity of all evaluated enzymes, except for GRed, which showed a significant decrease. A similar trend was observed for MIX, where all enzyme activities increased significantly, except GPx, which exhibited a significant decrease (Fig. 1). Notably, no significant changes in GSH levels were observed in *D. rerio* following exposure to TRIM, while a significant increase occurred after MIX exposure. The results suggest that TRIM and MIX induced the production of reactive oxygen species (ROS), activating the antioxidant defense system of *D. rerio*. However, this response was insufficient to prevent oxidative damage, as indicated by the significant increase in TBARS levels (Fig. 1). These contrasting responses between individual antibiotics and MIX suggest different types of interactions. In several biomarkers, the response observed in MIX was stronger than that of the individual compounds, indicating potential synergistic effects, particularly in oxidative stress and lipid peroxidation (Fig. 1). However, for specific enzymes such as GPx activity, the response in MIX was lower than expected based on individual exposures, suggesting possible antagonistic interactions (Fig. 1). These differences may be explained by the distinct modes of action of SMX and TRIM, which both interfere with the folate pathway but at different enzymatic steps, potentially leading to complex interactions at the biochemical level. Additionally, environmental factors such as increased temperature and pH may further modulate these interactions by altering metabolic rates, enzyme activity, and chemical bioavailability, ultimately influencing nature and magnitude of mixture effects.

The widespread presence of antibiotics in aquatic environments is a growing concern due to their potential adverse effects on aquatic organisms (Carvalho and Santos 2016). These contaminants can persist in the environment, accumulate in organisms, and interfere with essential metabolic and physiological processes. The toxicity of antibiotics to aquatic organisms tends to remain constant when water temperature and pH are within standard values, such as those used in laboratory tests (e.g., Bethke et al. 2023). However, studies on the antibiotics understudy have shown that different oxidative stress responses can occur, even when the same antibiotic and concentration are applied (e.g., Boreham et al. 2024). This variability leads to differing responses within the same species, emphasizing that the effects of antibiotics are not uniform. Such variability can be reflected by the organisms’ baseline metabolic and physiological states, reinforcing the relevance of using integrative assessments of an organism’s health, which allow for more robust interpretations and facilitate comparisons across different studies. For instance, several studies (e.g., Iftikhar & Hashmi 2021; Limbu et al. 2018; Liu et al. 2020) found that even at relatively low concentrations of SMX (< 1000 µg/L), different responses in antioxidant, detoxification, and inflammatory mechanisms were observed in various fish species, including *Oreochromis niloticus*,* D. rerio*, and *Cyprinus carpio*. Liu et al. (2020) further reported that exposure to SMX concentrations below 100 µg/L significantly increased ROS levels and CAT activity, while inducing immune and inflammatory responses in zebrafish embryos. In contrast, Tokanová et al. (2021) found that zebrafish exposed to SMX at concentrations of 50, 100, and 500 µg/L for 14 days exhibited no significant changes in GPx, GRed, and GSTs activities, nor TBARS levels, highlighting the variability in oxidative stress responses. In line with these findings, Diogo et al. (2025c) reported that at 26 °C, exposure of *D. rerio* to SMX (150 µg/L) triggered changes in antioxidant and detoxification mechanisms (with increased activities of SOD, GRed, and GSTs, and GSH levels; decrease in CAT and GPx activities), and caused lipid peroxidation. Similarly, observed that TRIM exposure (30 µg/L) led to pronounced suppression of antioxidant defenses, with no significant effects on CAT activity and TBARS levels, but a marked reduction in SOD, CAT, GRed, GPx, and GSTs activities, as well as decreased GSH levels (Diogo et al. 2025a). Diogo et al. (2025b) found that under neutral pH (7.5), 150 µg SMX/L caused significant activation of the antioxidant system, with increased activities of GPx, GSTs, and elevated GSH levels, while also inducing lipid peroxidation. In contrast, under the same pH conditions, 30 µg TRIM/L resulted in increased activities of CAT, GRed, and GSTs, along with higher GSH and TBARS levels, while decreasing SOD and GPx activities. These studies collectively highlight that both SMX and TRIM, at 26 °C and pH 7.5, not only induced significant changes in antioxidant and detoxification mechanisms but also led to DNA damage.

Regarding cholinergic neurotransmission, SMX exposure significantly increased AChE activity, whereas TRIM and MIX led to a decrease (Fig. 1). These findings align with previous studies on *D. rerio*, which linked AChE activity changes to the mechanisms of action of sulfonamides and TRIM (Crivello et al. 2010; Huo et al. 2023; Lee et al. 2012). Different studies have demonstrated that SMX and TRIM can significantly affect AChE activity in aquatic organisms, although the effects vary depending on concentration and exposure duration (e.g., Zhang et al. 2023). For instance, Diogo et al. (2024) observed a reduction in *Daphnia magna* AChE activity after 10 days of exposure to SMX (45 mg/L) and TRIM (above 3.1 mg/L), whereas lower SMX concentrations (2.8-45 mg/L) had no significant impact. Zhang et al. (2023) reported a decrease in AChE activity in the same species after 14 days of exposure to SMX (100 and 1000 μg/L), leading to impaired locomotion due to neurological damage. These findings support the idea that SMX and TRIM can interfere with cholinergic function in different aquatic organisms. The here-presented results show that environmentally relevant concentrations can cause biologically meaningful effects, especially when acting alongside climate-stressors such as temperature and pH. SMX and TRIM are antifolate drugs that disrupt folate synthesis, essential for DNA, RNA, and protein production. SMX inhibits dihydrofolic acid synthesis by mimicking para-aminobenzoic acid, while TRIM blocks dihydrofolate reductase, preventing tetrahydrofolate formation (Masters et al. 2003). Disruption of the folate pathway can impair nervous system development and neurotransmitter synthesis, contributing to neurotoxicity (Eichwald et al. 2023; Haruki et al. 2013; Parashar et al. 2016). According to Huo et al. (2023), sulfonamides can also interfere with neurotransmitter function and endocrine pathways by disrupting folate metabolism. Additionally, SMX and TRIM impair tetrahydrobiopterin (BH4) synthesis, a cofactor essential for producing neurotransmitters (like serotonin and dopamine). These disruptions may alter AChE activity, as reported in previous studies (e.g., Crivello et al. 2010), ultimately affecting neurotransmitter balance and neural function. Several antibiotics (including enrofloxacin, flumequine, norfloxacin, erythromycin, sulfadiazine, and sulfisoxazole) have been shown to impact AChE activity in aquatic organisms under standard temperature conditions (e.g., Liu et al. 2014; Oropesa et al. 2024; Tian et al. 2024; Yang et al. 2019). Such alterations can lead to behavioral changes and developmental impairments and are often associated with weakened defense mechanisms (Huo et al. 2023; Yan et al. 2016). For example, Yang et al. (2019) demonstrated that 500 µg/L SMX significantly reduced AChE activity in the brain tissue of the fish *Carassius auratus*, suggesting neurotoxic effects. Diogo et al. (2025a) reported that 150 μg SMX/L, 30 μg TRIM/L, and their mixture (the same concentrations tested in the present study) increased AChE activity in *D. magna.* Additionally, the same authors found that SMX significantly decreased AChE activity, while MIX led to a significant increase in zebrafish embryos after 96 h. These findings emphasize the complexity of antibiotic-induced neurotoxicity in aquatic organisms, revealing that the effects of individual antibiotics and their mixtures can vary depending on species, exposure conditions, and compensatory mechanisms. This variability also reflects the complexity of mixture interactions, where antibiotics may exert synergistic, additive, or antagonistic effects depending on their modes of action and the biological response considered. All these aspects underscore the need for further research to assess the environmental risks of antibiotic contamination, but also the importance of adopting consistent and integrative ecotoxicological approaches that enable more rigorous comparisons across studies.

A significant increase in LDH activity was observed after SMX exposure at the standard scenario, suggesting that SMX exposure potentially enhanced anaerobic metabolism, possibly as a compensatory response to increased metabolic demands (Fig. 1). The results showed a significant decrease in carbohydrate and lipid contents, along with a reduction in Ea and Ec, while protein content and CEA increased after SMX exposure (Fig. 1). These findings indicate a metabolic shift towards anaerobic energy production, driven by the increase in LDH activity and the reduction of Ea and Ec, as a response to SMX-induced stress. LDH plays a key role in regulating the final stage of the anaerobic glycolytic pathway, essential for carbohydrate metabolism, acting as the primary energy source, especially under stress conditions (Abdallah et al. 2024; Tasneem and Yasmeen 2020). The observed increase in LDH activity suggests an enhanced conversion of pyruvate to lactate, reflecting a shift from aerobic to anaerobic metabolism (Abdallah et al. 2024). This transition, commonly triggered by stress, leads to an accelerated depletion of carbohydrate reserves, reinforcing the observed metabolic adjustments. Diogo et al. (2025c) observed that under standard temperature and pH conditions, exposure to 150 µg SMX/L increased LDH activity and decreased carbohydrate content and Ea in zebrafish. These authors indicate that *D. rerio* may shift toward additional metabolic pathways, particularly anaerobic metabolism, to meet its energy demands and redistribute cellular energy towards prioritized, essential functions. In contrast, TRIM and MIX exposure did not lead to significant changes in LDH activity, carbohydrate content, or Ea, suggesting a different metabolic response compared to SMX. Instead, there was a decrease in Ec and an increase in lipid content and CEA (Fig. 1). These results indicate a possible shift in energy allocation, allowing the organism to maintain energy balance without relying on anaerobic metabolism. The unchanged LDH activity suggests that anaerobic metabolism was not significantly activated, while the decrease in Ec may reflect an overall reduction in energy demand, possibly as an adaptive response to the stressor. Abe et al. (2018) also report similar results in zebrafish exposed to dyes, resulting in a significant decrease in Ec, and CEA increased, reflecting an increase in metabolic activity and a redistribution of energy resources toward defense processes and cellular maintenance. Although a few studies have explored the effects of antibiotics on energy allocation, research has shown that contaminants (e.g., metals, effluents, and nutrients) can significantly impact the energy distribution of various aquatic organisms (e.g., microcrustaceans, bivalves, and fish) (e.g., Aderemi et al. 2018; Almeida et al. 2022; Gomes et al. 2016; Tourinho et al. 2022). These stressors can alter the balance between energy available and energy consumed, affecting key physiological processes. Disruptions in energy allocation can compromise growth and reproduction, reducing an organism’s ability to survive and adapt to environmental challenges.

**Fig. 2:**
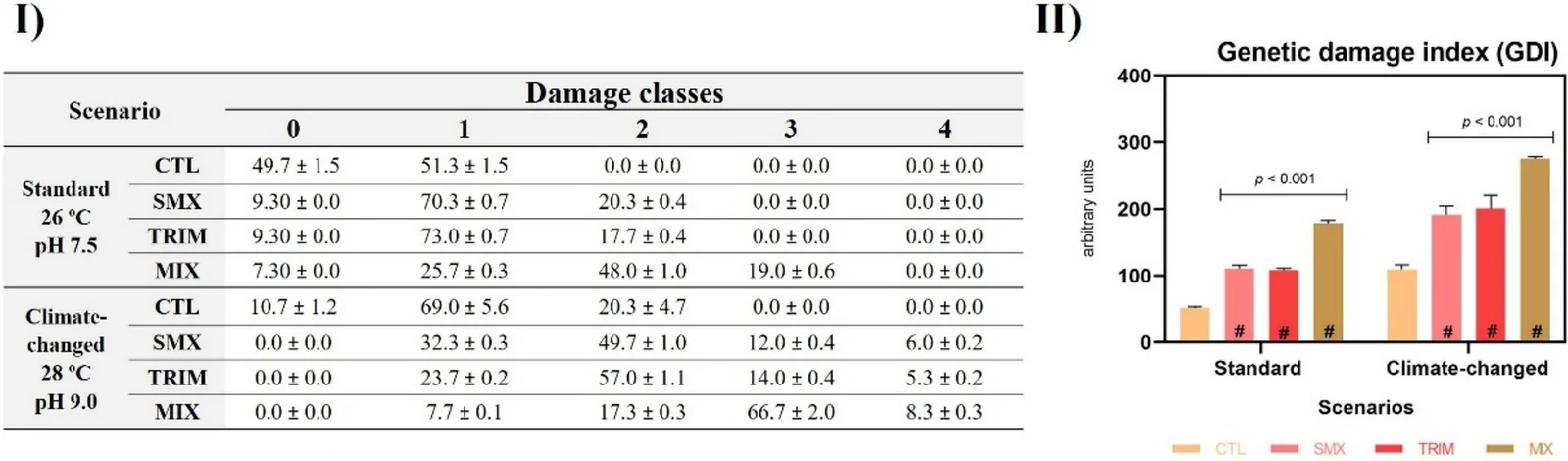
Results of the comet assay conducted in gills of *Danio rerio* after chronic exposure (28 days) to the control group (CTL, without antibiotics), sulfamethoxazole (150 µg SMX/L), trimethoprim (30 µg TRIM/L), and mixture (MIX = 150 µg SMX/L + 30 µg TRIM/L) under standard (26 °C and pH 7.5) and climate-changed (28 °C and pH 9.0) scenarios. **I**) Results of percentage of damage classes. **II**) Genetic Damage Index (GDI, expressed as arbitrary units). Data are expressed as mean ± standard error bars. Significant *p* levels correspond to the interaction effects between antibiotics and environmental scenarios tested (two-way ANOVA; Table S2). Number sign (#) indicates significant differences between the control group and antibiotic treatments (Dunnett’s test; *p* < 0.05)

The present study also demonstrated that all the antibiotic treatments caused a significant increase in the genetic damage index (Fig. 2II). However, SMX and TRIM caused considerable DNA damage, particularly in classes 1 and 2, while those exposed to MIX exhibited more severe damage, with DNA damage predominantly in classes 1, 2, and 3 (Fig. 2I and Table S2), suggesting that MIX enhanced genotoxicity beyond what would be expected from single-compound exposures. SMX and TRIM act as folic acid antagonists, disrupting DNA synthesis by inhibiting dihydrofolate reductase (Masters et al. 2003). In addition to promoting excessive ROS production, which increases oxidative stress and contributes to DNA damage (Fig. 2), SMX and TRIM further enhance genotoxic effects through their mode of action. As folic acid antagonists, these antibiotics inhibit dihydrofolate reductase, preventing the conversion of dihydrofolic acid to tetrahydrofolic acid, a crucial step in thymidine production (Masters et al. 2003; Sangurdekar et al. 2011). This disruption impairs DNA replication and repair, increasing the likelihood of mutations and genotoxic effects, which can ultimately lead to cell dysfunction and death (Abou-Eisha et al. 1999; Mason and Levesque 1996). The more severe DNA damage observed in organisms exposed to MIX compared to individual antibiotics suggests a potential synergistic interaction, with oxidative stress and nucleotide synthesis inhibition amplifying DNA damage and posing a significant risk to cellular integrity. Different studies have already reported the genotoxic effects of SMX and TRIM across various aquatic species (e.g., *Raphidocelis subcapitata*, *Mytilus edulis*, *Oncorhynchus mykiss,* and *D. rerio*) (Lacaze et al. 2015; Liu et al. 2014; Papis et al. 2011). However, these findings are mostly based on exposures to individual compounds, whereas the present study provides additional insight by evaluating their effects in an antibiotic mixture under environmentally relevant mixture scenarios.

### Climate-changed scenario

**Fig. 3:**
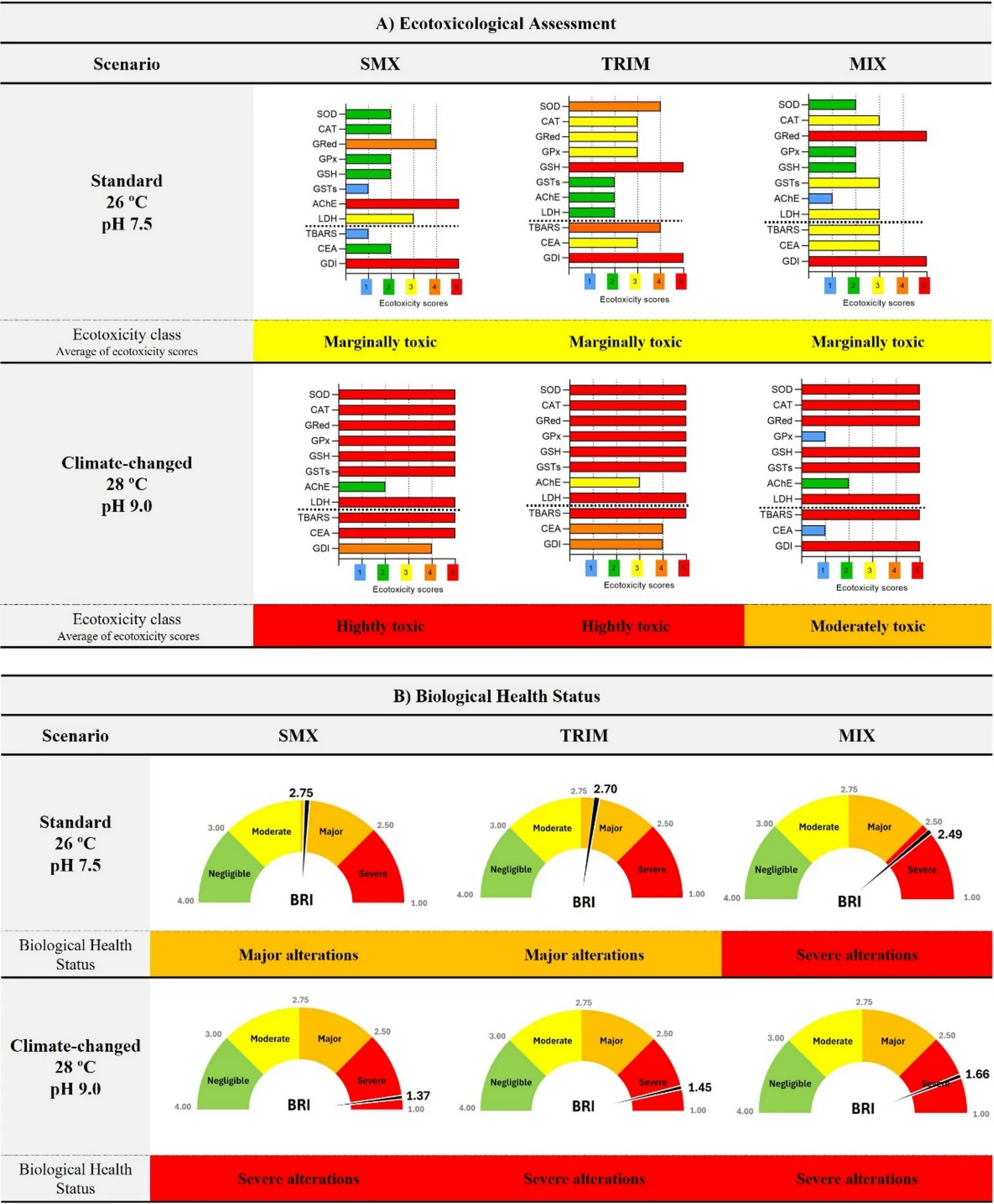
**A**) Results of ecotoxicity scores (ranging from 1 to 5) and the final toxicity classification (slightly toxic (ST - green), marginally toxic (MGT - yellow), moderately toxic (MT - orange), and highly toxic (HT - red); for additional information, *see* Table S2) determined for each antibiotic treatment under standard (26 °C and pH 7.5) and climate-changed (28 °C and pH 9.0) scenarios. **B**) Biomarker Response Index (BRI) and the corresponding classification of Biological Health Status were also presented. BRI values: 1.00 ≤ BRI ≤ 2.50, severe alterations (red); 2.51 ≤ BRI ≤ 2.75, major alterations (orange); 2.76 ≤ BRI ≤ 3.00, moderate alterations (yellow); 3.01 ≤ BRI ≤ 4.00, negligible alterations (green) (for additional information, *see* Table S2)

The results of all the biochemical markers evaluated after exposure to environmentally relevant concentrations of SMX, TRIM, and their MIX under changed temperature and pH scenario (28 °C and pH 9.0) are shown in Fig. 1. Significant interactions were observed between antibiotic concentrations and changed environmental factors (temperature and pH) for all evaluated parameters (Fig. 1; Table S2) for all the biochemical markers. Under the climate-changed scenario, all antibiotics induced a significant increase in all the antioxidant/detoxification mechanisms (SOD, CAT, GRed, GPx, and GSTs activities, GSH levels), and lipid peroxidation (TBARS levels, Fig. 1). Only GPx activity remained unaffected after exposure to MIX. These findings suggest that altered environmental conditions (increased temperature and pH), aligned with climate change projections for freshwater ecosystems, exacerbated oxidative stress in *D. rerio*, eliciting a broadly enhanced activation of antioxidant and detoxification pathways in response to all antibiotic exposures. Notably, the MIX exposure scenario often induced stronger responses than the individual antibiotics (Figs. 1, 2, and 3), suggesting potential synergistic interactions in activating antioxidant defenses under elevated temperature and pH. However, some enzymes (e.g., GPx in MIX, Figs. 1 and 3) did not respond proportionally, indicating endpoint-specific antagonistic or compensatory effects.

Multiple studies have reported that exposure to non-standard temperature and pH conditions enhances the toxicity of various chemicals, such as pharmaceuticals and pesticides, in aquatic organisms (Bethke et al. 2023). This increased toxicity can interfere with metabolic pathways and lead to physiological impairments (Kazmi et al. 2022; Macek et al. 1969; Pinheiro et al. 2021; Zhou et al. 2014). The organism’s response variations may be due to increased antibiotic toxicity or bioavailability, higher metabolic rates at elevated temperatures, and changes in enzyme efficiency caused by pH shifts (Pinheiro et al. 2021). These reasons likely contributed to activating stronger antioxidant defense mechanisms, indicating a greater oxidative stress (Dionísio et al. 2020). Supporting these findings, Diogo et al. (2025b) reported that under pH 9.0, both SMX and TRIM activated antioxidant defenses in *D. rerio* (e.g., increased CAT and GSTs activities). However, although lipid peroxidation was not observed in SMX-exposed organisms, this antibiotic still induced genotoxicity, whereas TRIM exposure resulted in oxidative damage. Notably, the combination of SMX and TRIM (MIX) had the most severe effects, disrupting antioxidant defenses and consequently leading to both lipid peroxidation and DNA damage (Diogo et al. 2025b). Similarly, Diogo et al. (2025c) found that SMX exposure at 28 °C enhanced antioxidant capacity in *D. rerio* but also increased oxidative damage. In contrast, TRIM exposure at the same temperature only reduced CAT activity while increasing GSTs activity in *D. rerio*. When exposed to MIX at 28 °C, the organism appeared to activate a more robust antioxidant defense, likely as an adaptive response to oxidative stress induced by elevated temperature.

The results of cholinergic neurotransmission after exposure to environmentally relevant concentrations of SMX, TRIM, and their MIX under a climate-changed scenario (28 °C and pH 9.0) are shown in Fig. 1. All antibiotic treatments induced a significant increase in AChE activity (Fig. 1), with the greatest effect observed for MIX, highlighting the complexity of mixture effects, and the potential additive or synergistic interactions. This suggests that environmental factors influence how antibiotics interact with enzyme activity, causing a shift in their effects (Fig. 1). The combined impact of temperature and pH likely intensified the neurotoxic effects of the antibiotics, leading to the observed rise in AChE activity. Higher temperatures accelerate metabolic processes, which may trigger compensatory mechanisms, as reported by Diogo et al. (2025c), who found that temperature significantly affects basal levels of AChE activity in *D. rerio*, with notable variations observed at different temperatures (26, 28, and 32 °C). Furthermore, the increase in AChE activity following antibiotic exposure at elevated temperatures likely reflects a complex interaction between thermal stress, neurotoxicity, and compensatory responses. Changes in pH can also significantly influence AChE activity by altering the enzyme’s stability, structure, and function (Marinho et al. 2019). Additionally, pH fluctuations affect the ionization and bioavailability of antibiotics, potentially modifying their interaction with AChE (as changes in pH can alter the chemical form of the compound -e.g., neutral or charged- which may influence its ability to cross biological membranes or bind to the enzyme’s active site) (Marinho et al. 2019; Serova et al. 2020). This highlights the importance of maintaining pH balance for preserving nervous system integrity and overall physiological stability (Komersová et al. 2018; Serova et al. 2020). Consequently, shifts in pH may either amplify or mitigate the neurotoxic effects of antibiotics, further influencing AChE activity and neurotransmitter regulation. Marinho et al. (2019) investigated the combined effects of different temperatures (18, 22, 26, and 30 °C) and pH (5.0, 6.0, 7.0, and 8.0) on AChE activity in zebrafish, demonstrating that higher temperature and pH accelerated metabolic reactions and altered the enzyme’s structure, which likely triggered a compensatory response in the fish’s nervous system. These findings underscore how environmental changes can affect enzyme function and neurophysiological health in fish (Komersová et al. 2018; Marinho et al. 2019; Serova et al. 2020), with these effects potentially being exacerbated by antibiotic exposure. As climate change continues to intensify temperature and pH fluctuations, the combined effects of these abiotic factors and antibiotic exposure may lead to unpredictable neurotoxic outcomes. These interactions could disrupt neurotransmission and impair key physiological functions such as locomotion, feeding, escape responses, and reproduction, ultimately affecting the fitness, survival, and overall health of aquatic species (Muñoz-Peñuela et al. 2022).

The exposure to SMX, TRIM, and MIX under elevated pH and temperature conditions led to notable shifts in metabolic responses, with each antibiotic affecting metabolic pathways differently (Fig. 1). A significant increase in LDH activity occurred after SMX exposure, whereas TRIM and MIX resulted in a significant decrease (Fig. 1 and Table S2). This suggests that SMX exposure potentially enhanced anaerobic metabolism, possibly as a compensatory response to increased metabolic demands under antibiotic and changed conditions. In contrast, the reduction in LDH activity observed with TRIM and MIX, under climate-changed conditions, suggests a lower demand for anaerobic metabolism or a possible toxic effect that impairs enzymatic capacity (Fromm 1975; Kirk 2023). Although no studies have reported the combined effects of temperature, pH, and antibiotics on LDH activity, literature has demonstrated the influence of these factors individually (e.g., Kirk (2023) and Zakhartsev et al., (2004)). LDH, a key enzyme in anaerobic metabolism, is highly temperature-dependent, with studies showing increased activity at elevated temperatures (Farhana and Lappin 2024; Zakhartsev et al. 2004). Schnurr et al. (2013) reported a significant rise in zebrafish LDH activity at 32 °C, suggesting that temperature shifts can alter energy metabolism. Additionally, Diogo et al. (2025b) reported LDH activation in *D. rerio* exposed to SMX (150 μg/L), TRIM (30 μg/L), and their MIX at neutral and alkaline pH (7.5 and 9.0), suggesting that zebrafish may rely more on anaerobic pathways to sustain cellular functions under such conditions. These findings highlight the potential for rising temperatures and pH fluctuations to exacerbate antibiotic-induced metabolic stress, with consequences for the energy balance of aquatic organisms.

Regarding the energy reserves, SMX and TRIM exposure both led to significant increases in carbohydrate and lipid content, as well as Ea and CEA (Fig. 1 and Table S2), suggesting a shift toward energy storage and allocation as a compensatory mechanism to counteract toxic stress (Abe et al. 2018; Chiang 2014). This response likely helps maintain cellular homeostasis under temperature and pH fluctuations. However, while Ec remained unchanged after SMX exposure, it significantly increased after TRIM exposure, indicating higher energy expenditure (Fig. 1). This suggests that TRIM may impose greater metabolic demands, requiring organisms to balance both energy storage and consumption. In contrast, MIX exposure decreased carbohydrate content, Ea, and CEA while increasing lipid content and Ec, suggesting a reduction in overall energy reserves alongside rising metabolic expenditure (Fig. 1). The increase in lipid content following MIX exposure suggests a disruption in lipid metabolism, potentially linked to stress responses. Similar effects have been observed in previous studies (e.g., Lei et al. 2023; Park et al. 2020), where antibiotics and other contaminants altered lipid storage and mobilization. These changes may result from oxidative stress, energy redirection toward detoxification, or altered metabolic regulation. Additionally, the rise in Ec indicates heightened metabolic activity, potentially reflecting increased energy demands to cope with environmental stress. This is further supported by the significant activation of antioxidant and detoxification mechanisms (SOD, CAT, GRed, GPx and GSTs activities and GSH levels; Fig. 1) observed, suggesting that the organisms are mobilizing additional energy resources to counteract oxidative damage induced by antibiotic exposure, temperature, and pH changes. Similar results were reported by Aderemi et al. (2018) in *Raphidocelis subcapitata* after exposure to various antibiotics (sulfamethoxazole, erythromycin, clarithromycin, and ciprofloxacin), where a significant increase in Ec was accompanied by elevated SOD activity, indicating an oxidative stress response associated with higher energy consumption. Additionally, these authors reported a decline in CEA, due to a marked difference between Ea and Ec, suggesting a shift in the organism’s energy budget toward coping with stress (Aderemi et al. 2018). Environmental factors, such as temperature and pH variations, are also known to impact metabolic processes. Anacleto et al. (2018) highlighted those warming force organisms to adjust their metabolism, often increasing energetic costs and compromising energy allocation for essential functions. Similarly, Bolner et al. (2014) observed that alkaline conditions increased Na⁺/K⁺-ATPase activity in the gills and kidneys of fish *Rhamdia quelen*, indicating a greater energy investment in ion regulation. Since this enzyme is ATP-dependent, its upregulation suggests an increased metabolic demand to maintain ionic homeostasis under elevated pH (Bolner et al. 2014). Overall, these findings support the idea that environmental stressors, and antibiotic contamination, can further disrupt energy homeostasis, increasing metabolic costs associated with detoxification, repair, and homeostasis. This reallocation of energy often occurs at the expense of growth, reproduction, and other vital functions, ultimately compromising organismal fitness and survival (Bethke et al. 2023; Jesus et al. 2018).

Exposure to antibiotics has been widely reported to induce DNA damage, raising concerns about their potential impact on aquatic organisms (Papis et al. 2011; Ramesh et al. 2018; Sharma et al. 2021), namely regarding health, reproductive success, and survival. However, environmental factors like temperature and pH fluctuations also play a significant role in DNA integrity (regarding CTL results between scenarios). A significant increase in genetic damage index was observed after exposure to all the antibiotics (Fig. 2); however, organisms exposed to SMX and TRIM exhibited less severe DNA damage (higher percentage damage class 2) than MIX (higher percentage damage class 3). This more severe DNA damage observed after MIX (Fig. 2) exposure indicates a potential synergistic interaction between SMX and TRIM under climate-changed conditions, exceeding the genotoxic effects observed for individual antibiotics. Diogo et al. (2025c) reported a significant rise in DNA damage at varying temperatures (26, 28, and 32 °C), suggesting that under global warming conditions, the combined effects of SMX and TRIM could present a greater genetic risk to *D. rerio* than exposure to each antibiotic individually. Similarly, Diogo et al. (2025b) demonstrated that genotoxicity occurred consistently after exposure to SMX, TRIM, and MIX, regardless of pH (6.5, 7.5, and 9.0). Although *D. rerio* may activate antioxidant and detoxification mechanisms to counteract ROS production, these defense and detoxification mechanisms can be effective in preventing lipid peroxidation but not DNA damage. This suggests that these cellular responses prioritized membrane protection over nuclear integrity, leaving DNA more vulnerable to genotoxic effects. Overall, the interplay between antibiotic exposure and abiotic factors like temperature and pH fluctuations may amplify genetic risks in aquatic organisms, reinforcing the need for comprehensive environmental risk assessments that account for both chemical pollution and climate change, and evaluating different biological responses of organisms.

### Beyond biomarkers: integrative assessment of organism’s health under environmental stressors

The integration of multiple ecotoxicological assessment tools, including the toxicity classification (ecotoxicological assessment, Fig. 3A) and biological health status (Fig. 3B), provided a more comprehensive evaluation of the impacts of antibiotic exposure on *Danio rerio* under different environmental scenarios. These approaches allowed for multidimensional analysis, capturing both the biochemical and physiological responses of fish to SMX, TRIM, and their MIX, as well as the overall severity of toxicity induced by different abiotic scenarios (increased temperature and pH). The results showed that SMX, TRIM, and their MIX, under the standard scenario, were marginally toxic to *D. rerio* (Fig. 3A; e.g., antioxidant defenses and DNA damage alterations), but according to biological health status, SMX and TRIM still caused major alterations, while MIX caused severe alterations (Fig. 3B). In contrast, under a climate-changed scenario, SMX and TRIM were highly toxic, while MIX was moderately toxic (Fig. 3A; e.g., antioxidant defenses and energy metabolism disruptions, and increased lipid peroxidation and DNA integrity). However, all antibiotic treatments caused severe alterations in *D. rerio* (Fig. 3B). These results suggest that altered environmental conditions, such as increased temperature and pH, intensify the toxicity of antibiotics, both individually and in mixtures, leading to more severe physiological disruptions in *D. rerio*. However, under these climate-changed conditions, individual antibiotics (SMX and TRIM) were more toxic than their mixture. This could indicate a potential interactive effect within the MIX, where combined exposure triggers biological responses that slightly mitigate toxicity compared to individual exposures, possibly through the activation of compensatory or adaptive mechanisms. A possible hypothesis for the greater toxicity of individual antibiotics can be associated with the occurrence of antagonistic or non-additive interactions within the mixture. When combined, SMX and TRIM may interfere with each other’s absorption, metabolism, or target binding, potentially reducing their individual toxic effects. Moreover, co-exposure may induce stronger compensatory responses in *D. rerio*, such as enhanced activation of detoxification pathways or stress tolerance mechanisms, which are less pronounced during single-compound exposures. These interactions are especially relevant under elevated temperature and pH conditions, which can modulate chemical bioavailability, membrane permeability, and enzymatic activity, ultimately influencing toxicity outcomes. Regardless, the increased toxicity observed under climate-changed scenario highlights the critical role of environmental factors in shaping contaminant effects and underscores the heightened risk posed by antibiotics in a changing climate. This integrative ecotoxicological assessment improves the overall understanding of the effects of antibiotic exposure under varying environmental conditions and enables more accurate and calibrated comparisons between studies by incorporating both biochemical and physiological endpoints and accounting for the influence of key abiotic factors.

## Concluding remarks

This study demonstrates that antibiotic toxicity is strongly modulated by environmental conditions, with increased temperature and pH significantly amplifying adverse effects on aquatic organisms. Notably, biologically meaningful alterations were observed even at environmentally relevant concentrations, indicating that current risk assessments based on standard conditions may underestimate the ecological impact of these contaminants. The results also reveal that mixture effects are complex and context-dependent, with interactions shifting between synergistic and antagonistic scenarios depending on the biological endpoint and environmental factors. Under climate change–related conditions, these interactions led to more pronounced disruptions to oxidative balance, energy metabolism, and DNA integrity, ultimately compromising organism health. Overall, this study highlights the need to incorporate environmental factors such as temperature and pH, as well as mixture toxicity, into ecotoxicological assessments. Failure to do so may lead to an underestimation of the risks posed by antibiotics and other contaminants under future climate scenarios, with potential consequences for aquatic ecosystem stability and biodiversity.

The cumulative impact of these stressors (contaminants and environmental factors, such as temperature and pH alterations) extends beyond individual organisms, affecting biodiversity, food web stability, and overall ecosystem resilience. Understanding how environmental changes modulate contaminant toxicity is crucial for the development of effective regulatory frameworks and conservation strategies. Without appropriate intervention, the combined pressures of abiotic factors and antibiotic pollution could lead to lasting ecological imbalances, ultimately threatening the functional stability of aquatic ecosystems. Implementing policies that limit the indiscriminate use of antibiotics, safeguard aquatic habitats, and promote sustainable water resource management is crucial to prevent long-term ecological imbalances. A proactive, multidisciplinary, and integrative approach is essential to mitigate these environmental pressures and ensure the long-term conservation and resilience of aquatic ecosystems.

## Supplementary Information

Below is the link to the electronic supplementary material.

Supplementary file1 (DOCX 35.1 kb)

## Data Availability

All generated data are presented in the manuscript and supplementary files.
