# Supplementary material for: Environmental assessment of antibiotic toxicity under climate change–related temperature and pH scenarios using Danio rerio
Source: Fish Physiol Biochem. 2026 Jul 31;52(4):133. doi: 10.1007/s10695-026-01750-9 (PMC13427809; doi:10.1007/s10695-026-01750-9)
Supplement: Supplementary file 1 — Supplementary file1 (DOCX 35.1 kb) [file 10695_2026_1750_MOESM1_ESM.docx]

**Table S1.** Summary details of the methodology for the biomarker determinations performed herein.

| **Biomarkers** | | | **Biomarker determinations** | | |
| --- | --- | --- | --- | --- | --- |
|  |  |  | **Spectrophotometric readings (nm)** | **Result expression units** | **References** |
| Superoxide dismutase activity (SOD) | | | 500 | units min/mg/protein | Diogo et al. (2025b) |
| Catalase activity (CAT) | | | 240 | mmol/min/mg protein |  |
| Glutathione Peroxidase activity (GPx) | | | 340 | mmol/min/mg protein |  |
| Glutathione Reductase activity (GRed) | | | 340 | mmol/min/mg protein |  |
| Glutathione content (GSH) | | | 412 | µg/mg protein |  |
| Glutathione S-Transferases activity (GSTs) | | | 340 | mmol/min/mg protein |  |
| Thiobarbituric Acid Reactive Substances levels (TBARS) | | | 535 | mmol/mg protein |  |
| Cellular Energy Allocation (CEA) | Available Energy (Ea) | Carbohydrates (CARBO) | 492 | mJ/mg fresh weight |  |
|  |  | Lipids (LIP) | * |  |  |
|  |  | Protein (PROT)** | 595 |  |  |
|  | Energy consumed (Ec) | Electron Transport System (ETS) | 490 | mJ/mg fresh weight/min |  |
| Lactate dehydrogenase activity (LDH) | | | 340 | mmol/min/mg protein |  |
| Acetylcholinesterase activity (AChE) | | | 412 | mmol/min/mg protein |  |
| *Extraction procedure through the biphasic solvent system consisting of chloroform/methanol/water. The results (% of lipids) were obtained by the difference between the weight of tubes before and after lipid extraction.  **Protein content was used as one of the parameters to estimate the available energy in CEA calculations and to normalize the biomarkers results (except CEA biomarkers). | | | | |  |

**Table S2.** Summary table of the two-way ANOVA applied to abiotic environmental factors (AEF; corresponding to pH and temperature variations) and antibiotics treatment. Bold values stand for significant differences between tested factors (pH + temp *vs* antibiotics) (*p* < 0.05).

| **Endpoint** | **Source variation** | **d.f.** | **F** | ***p*** |
| --- | --- | --- | --- | --- |
| Specific growth rate | AEF | 1 | 0.979 | 0.337 |
|  | Antibiotics | 3 | 2.416 | 0.104 |
|  | AEF x Antibiotics | 3 | 0.598 | 0.625 |
|  | Residual | 16 |  |  |
| SOD activity | AEF | 1 | 75.974 | **< 0.001** |
|  | Antibiotics | 3 | 76.789 | **< 0.001** |
|  | AEF x Antibiotics | 3 | 42.790 | **< 0.001** |
|  | Residual | 16 |  |  |
| CAT activity | AEF | 1 | 144.986 | **< 0.001** |
|  | Antibiotics | 3 | 215.754 | **< 0.001** |
|  | AEF x Antibiotics | 3 | 32.804 | **< 0.001** |
|  | Residual | 16 |  |  |
| GRed activity | AEF | 1 | 394.954 | **< 0.001** |
|  | Antibiotics | 3 | 2109.409 | **< 0.001** |
|  | AEF x Antibiotics | 3 | 352.199 | **< 0.001** |
|  | Residual | 16 |  |  |
| GPx activity | AEF | 1 | 536.361 | **< 0.001** |
|  | Antibiotics | 3 | 377.458 | **< 0.001** |
|  | AEF x Antibiotics | 3 | 103.167 | **< 0.001** |
|  | Residual | 16 |  |  |
| GSTs activity | AEF | 1 | 222.293 | **< 0.001** |
|  | Antibiotics | 3 | 132.840 | **< 0.001** |
|  | AEF x Antibiotics | 3 | 52.826 | **< 0.001** |
|  | Residual | 16 |  |  |
| GSH content | AEF | 1 | 90.202 | **< 0.001** |
|  | Antibiotics | 3 | 48.958 | **< 0.001** |
|  | AEF x Antibiotics | 3 | 15.395 | **< 0.001** |
|  | Residual | 16 |  |  |
| TBARS levels | AEF | 1 | 147.951 | **< 0.001** |
|  | Antibiotics | 3 | 98.085 | **< 0.001** |
|  | AEF x Antibiotics | 3 | 53.910 | **< 0.001** |
|  | Residual | 16 |  |  |
| AChE activity | AEF | 1 | 1.131 | 0.303 |
|  | Antibiotics | 3 | 110.747 | **< 0.001** |
|  | AEF x Antibiotics | 3 | 248.492 | **< 0.001** |
|  | Residual | 16 |  |  |
| LDH activity | AEF | 1 | 1363.226 | **< 0.001** |
|  | Antibiotics | 3 | 433.544 | **< 0.001** |
|  | AEF x Antibiotics | 3 | 117.757 | **< 0.001** |
|  | Residual | 16 |  |  |
| CARBO content | AEF | 1 | 121.321 | **< 0.001** |
|  | Antibiotics | 3 | 298.548 | **< 0.001** |
|  | AEF x Antibiotics | 3 | 864.869 | **< 0.001** |
|  | Residual | 16 |  |  |
| PROT content | AEF | 1 | 49.069 | **0.045** |
|  | Antibiotics | 3 | 9.659 | **< 0.001** |
|  | AEF x Antibiotics | 3 | 20.485 | **0.046** |
|  | Residual | 16 |  |  |
| LIP content | AEF | 1 | 19.583 | **< 0.001** |
|  | Antibiotics | 3 | 239.344 | **< 0.001** |
|  | AEF x Antibiotics | 3 | 175.029 | **< 0.001** |
|  | Residual | 16 |  |  |
| Available energy (Ea) | AEF | 1 | 121.344 | **< 0.001** |
|  | Antibiotics | 3 | 298.567 | **< 0.001** |
|  | AEF x Antibiotics | 3 | 864.902 | **< 0.001** |
|  | Residual | 16 |  |  |
| ETS activity/  Energy consumed (Ec) | AEF | 1 | 112.571 | **< 0.001** |
|  | Antibiotics | 3 | 65.842 | **< 0.001** |
|  | AEF x Antibiotics | 3 | 114.318 | **< 0.001** |
|  | Residual | 16 |  |  |
| CEA | AEF | 1 | 38.024 | 0.096 |
|  | Antibiotics | 3 | 51.752 | **< 0.001** |
|  | AEF x Antibiotics | 3 | 763.675 | **< 0.001** |
|  | Residual | 16 |  |  |
| GDI | AEF | 1 | 456.611 | **< 0.001** |
|  | Antibiotics | 3 | 247.398 | **< 0.001** |
|  | AEF x Antibiotics | 3 | 5.176 | **0.011** |
|  | Residual | 16 |  |  |

**Table S3.** Ecotoxicity scores (1 to 5) and ecotoxicological classes established considering each parameter's effect percentage (*adapted from* Rodrigues et al., 2022).

| **Parameters** | **Percentage of effects** | | | | |
| --- | --- | --- | --- | --- | --- |
| SOD activity  CAT activity  GRed activity  GPx activity  GSH content  GSTs activity  AChE activity  LDH content | ]-5, 5[ | [-5, -30[  [5, 30[ | [-30, -60[  [30, 60[ | [-60, -90[  [60, 90[ | ≤ -90  ≥ 90 |
| TBARS levels  CEA  GDI | ]-∞,5[ | [5, 30[ | [30, 60[ | [60, 90[ | ≥ 90 |
| **Ecotoxicity scores** | **1** | **2** | **3** | **4** | **5** |
| **Ecotoxicity classes** | **Non toxic** | **Slightly toxic** | **Marginally toxic** | **Moderately toxic** | **Hightly toxic** |

**Table S4.** Biological health status of *D. rerio* was established based on the percentage of effects and BRI (*adapted from* Piva et al. (2011) and Li et al. (2019)) after exposure to the environmentally relevant concentration of SMX, TRIM, and MIX in standard and climate-changed scenarios.

| **Biomarker Response Index (BRI)** | | | | | |
| --- | --- | --- | --- | --- | --- |
| **Alterations percentage** | ≥ 100  $Sn$ = 1 | 50-100  $Sn=$2 | 19-49  $Sn=$3 | ≤ 18  $Sn=$4 |  |
| **BRI**  **=**  $\frac{\sum(Snx Wn)}{\sum Wn}$  $Sn$is the score^(1)^ and $Wn$ the relevance factor^(2)^ of each biomarker $n$ | 1.00 - 2.50 | 2.51 - 2.75 | 2.76 - 3.00 | 3.01 - 4.00 |  |
| **Biological health status** | **Severe**  **alterations** | **Major**  **alterations** | **Moderate alterations** | **Negligible alterations** |  |
| ^(1)^ $Sn$obtained according to the alterations percentage  ^(2)^ according to Piva et al. (2011) | | | | |  |
